# Dimensional Changes in Lipid Rafts from Human Brain Cortex Associated to Development of Alzheimer’s Disease. Predictions from an Agent-Based Mathematical Model

**DOI:** 10.3390/ijms222212181

**Published:** 2021-11-10

**Authors:** Guido Santos, Mario Díaz

**Affiliations:** 1 Systems Biology and Mathematical Modelling Group, Department of Biochemistry, Microbiology, Cell Biology and Genetics, Biology Section, Science School, Universidad de La Laguna, 38200 San Cristóbal de La Laguna, Spain; 2 Laboratory of Membrane Physiology and Biophysics, Department of Animal Biology, Edaphology and Geology, Biology Section, Science School, Universidad de La Laguna, 38200 San Cristóbal de La Laguna, Spain; madiaz@ull.es; 3 IUETSP (Instituto Universitario de Enfermedades Tropicales y Salud Pública de Canarias), Universidad de La Laguna, 38200 San Cristóbal de La Laguna, Spain

**Keywords:** Alzheimer disease, Braak stages, neuropathology, lipid rafts, lipid composition, mathematical modeling, agent-based model

## Abstract

Alzheimer’s disease (AD) is a neurodegenerative disease caused by abnormal functioning of critical physiological processes in nerve cells and aberrant accumulation of protein aggregates in the brain. The initial cause remains elusive—the only unquestionable risk factor for the most frequent variant of the disease is age. Lipid rafts are microdomains present in nerve cell membranes and they are known to play a significant role in the generation of hallmark proteinopathies associated to AD, namely senile plaques, formed by aggregates of amyloid β peptides. Recent studies have demonstrated that human brain cortex lipid rafts are altered during early neuropathological phases of AD as defined by Braak and Braak staging. The lipid composition and physical properties of these domains appear altered even before clinical symptoms are detected. Here, we use a coarse grain molecular dynamics mathematical model to predict the dimensional evolution of these domains using the experimental data reported by our group in human frontal cortex. The model predicts significant size and frequency changes which are detectable at the earliest neuropathological stage (ADI/II) of Alzheimer’s disease. Simulations reveal a lower number and a larger size in lipid rafts from ADV/VI, the most advanced stage of AD. Paralleling these changes, the predictions also indicate that non-rafts domains undergo simultaneous alterations in membrane peroxidability, which support a link between oxidative stress and AD progression. These synergistic changes in lipid rafts dimensions and non-rafts peroxidability are likely to become part of a positive feedback loop linked to an irreversible amyloid burden and neuronal death during the evolution of AD neuropathology.

## 1. Introduction

Alzheimer’s disease (AD) is the main cause of dementia. At present this disease has reached proportions of pandemic and it is expected to keep increasing incidence during the following decades [1]. In the current scenario of absence of efficient therapies, it becomes one of the main clinical challenges for this century. There is a genetic form of AD, named familial AD (FAD) representing less than 1% of total AD cases worldwide, the rest of AD cases belong to the pathological group known as late-onset AD (LOAD). Despite decades of research on the etiology of LOAD, the original cause of the disease development is still unknown, and the only solidly established risk factor for AD is aging [2]. Neuropathological hallmarks of AD include proteinopathies causing amyloid plaques, by aggregation of insoluble amyloid β peptides, and neurofibrillary tangles, by hyperphosphorylation of tau protein, both of which evolve with disease progression. The mechanisms of formation of extracellular β-amyloid accumulations and the formation of intracellular neurofibrillary tangles have received considerable attention and current knowledge supports the widely accepted amyloid cascade hypothesis [3,4]. It is accepted that these neuropathological features can explain the symptoms and the evolution of the pathology in late stages of sporadic LOAD and also in the genetic form which causes FAD, though there exist considerable differences in the development of both forms. However, the initial factors, processes and mechanisms that ultimately trigger the appearance of these proteinopathies in the brain of LOAD remain elusive.

A recent paradigm which partly explains the early phases of AD is based on changes in the lipid cell membrane composition of brain tissue. Under this paradigm, a specific subset of membrane microdomain, known as lipid rafts, play a central role on multiple facets of this early unbalance [5]. These lipid domains provide the favorable environment for lipid-protein interactions essential to act as signaling hubs condensing highly relevant proteins for nerve cell physiology [6,7], but also for neuropathological states, such as APP (amyloid precursor protein), the obligatory substrate for the production of amyloid β peptides [8,9].

Further to global changes in nerve cell membrane physical properties and lipid composition, cumulative evidence has shown that alterations in specific lipids particularly enriched in brain microdomains take an active role on controlling the neurodegeneration [5,9,10]. Of outmost relevance is docosahexaenoic acid (DHA), a long chain polyunsaturated acid fatty, present in high concentrations in brain cell membranes, but asymmetrically distributed between membrane domains. Indeed, current estimations indicate that although lipid rafts are liquid-ordered highly saturated lipid domains, they contain significant amounts of DHA, likely playing a role in fine tuning of intradomain mobility and interdomain boundaries [11,12]. Indeed, DHA is about 4–5 fold less abundant in lipid rafts than in non-rafts domains [13,14,15], and its depletion has been observed to actively participate in AD-type neurodegeneration in different animal and cellular models, as well as in the evolution of sporadic AD [16,17,18]. Further, epidemiological evidence supports a role of DHA in neuroprotection against AD [19,20]. However, the way this fatty acid is expected to delay the onset of the disease is largely due to its pleiotropic actions: from structural components in membrane phospholipids, modulator of synapto- and neurogenesis, to genetic regulation of antioxidant defense, amongst other multiple roles [5,21,22].

Based on this evidence, we have developed a grain coarse mathematical model to simulate the formation of microdomains based on the lipid composition of APP/PS1 transgenic mice, a widely used animal model for familial AD carrying mutated human forms of Amyloid Precursor Protein and Presenilin-1, which has been thoroughly studied in the context of this study [5,12,13,23,24]. The mathematical model was constructed as an agent-based model composed by elements that interact and interchange positions simulating the lipid dynamics within cell membranes. Agent-based models represent a strategy to reduce the scale of molecular dynamics simulations through coarse graining of the structure of lipid molecules. Similar approaches using this strategy with different scaling levels have been reported. For example, a coarse-grained simulation of the lipid membrane has been used to study the spontaneous separation of elements in a mix of cholesterol, saturates and unsaturated fatty acids [25]. Other studies have been performed to create molecular dynamics simulations of cell membranes reducing the atomic scale by coarse graining the elements [26,27,28,29]. If the simulations require larger models or consideration of additional elements, such as signal transduction, the process of coarse graining can lead to a cellular automata kind of simulation [30]. In this sense, our current work may be ascribed to a cellular automata simulation.

Our previous work in a transgenic model of AD (APP/PS1) was the first modeling approach that simulated the formation of lipid rafts using experimental measurements from in vivo brain tissue. In this animal model, we observed that lipid domains increased in size paralleling the evolution of neuropathological hallmark of the disease, namely amyloid plaques [24]. This increase in lipid rafts supports the lipid raft hypothesis of AD by contributing with another factor form microdomains to increase the production of β-amyloid, as wider regions would enhance the accumulation of amyloidogenic components of APP processing [3,4].

Here we have adapted the mathematical model to simulate the formation of lipid domains using lipid composition of lipid rafts from human brains in the different stages of AD progression according to the Braak and Braak stages [31]. The objective of the present study was the assessment and validation of the lipid raft size hypothesis proposed for APP/PS1 mice to human LOAD, using the actual lipid composition reported for brain cortex microdomains [11,14,15].

## 2. Results

### 2.1. Lipid Classes Composition of Cell Membranes and Lipid Rafts in Human Frontal Cortex

Neurochemical profiles of lipid classes and fatty acids in cell membranes, lipid rafts and non-rafts fractions from different stages of AD progression —were reported by our group in recent publications [11,14,15]. A condensed summary of the lipid classes’ contents from these experimental results is shown in Table 1. The amounts are expressed in percentage of total lipids. The expected asymmetry can be observed in the contents of n-3 long-chain polyunsaturated fatty acids (LCPUFA, mostly represented by DHA), n-6 LCPUFA (mainly arachidonic acid), saturated fatty acids and sphingolipids, between lipid rafts (A) and whole membranes (B) in Controls and at Braak and Braak stages ADI/II, ADIII/IV and ADV/VI. Changes of lipid classes associated to the disease in lipid rafts include: reduction in cholesterol, DHA and sphingolipids and increase in sterol esters and n-6 LCPUFA, in particular at initial and intermediate stages of AD. In the most advanced stage, ADV/VI, lipid rafts also contain reduced levels of DHA and monoenes, as well as higher levels of n-6 LCPUFA, sterol esters and saturates Further, in this stage, whole membranes contain significantly lower levels of cholesterol, DHA and n-6 LCPUFA as well as higher levels of sterol esters and saturates.

We have used these experimental measurements to validate the mathematical model for humans. We will introduce as an input for the model the lipid composition of frontal cortex cell membrane as an input for the model (Table 1B) and we will compare the output predicted by the model for the lipid composition of lipid rafts with the frontal cortex lipid raft composition (Table 1A).

### 2.2. Simulation of Cell Membrane Domains during the Evolution of AD

**Figure 1:**
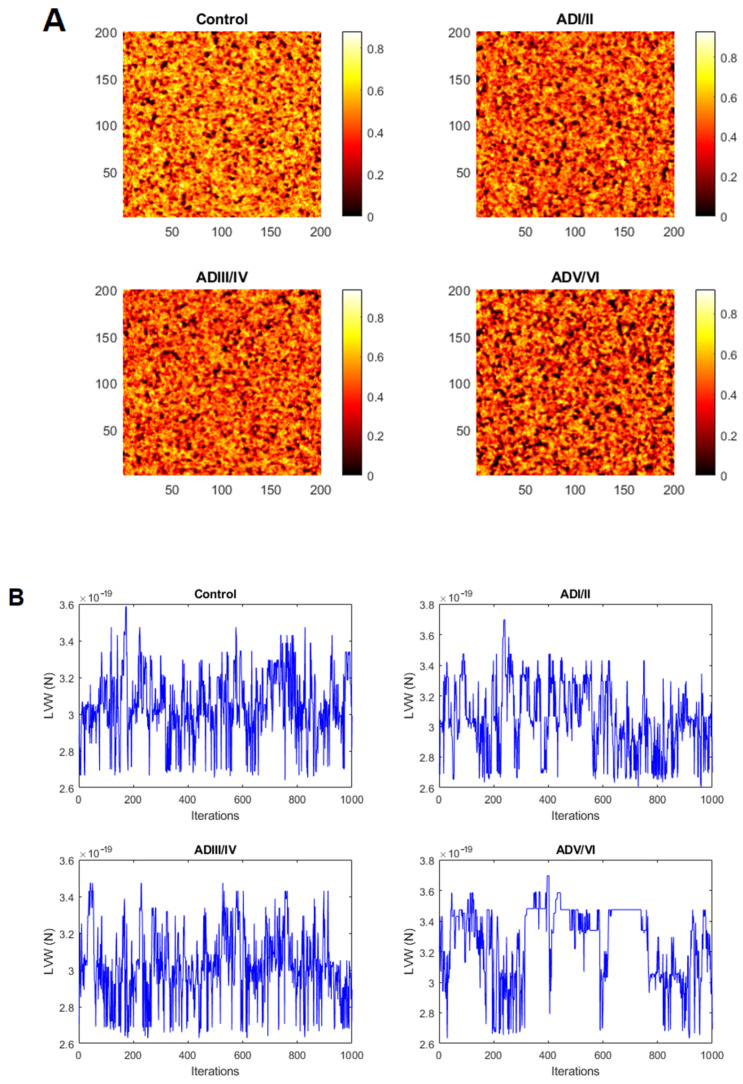
(**A**). Snapshots of a simulation after 1000 iterations in the four neuropathological stages. Color scale represents the mobility as the proportion of elements interchanged over the number of iterations. (**B**). Root-mean-square deviation (RMSD) for LVW forces (in N) corresponding to simulations displayed in panel A.

Following the mobility of the elements of the model one can identify a set of regions with very low mobility respecting the surrounding area that we correlate with lipid rafts in our membrane model (Figure 1). A first glimpse at the images in Figure 1 already suggests differences on the frequency and shapes of the simulated rafts between the four neuropathological conditions predicted by the model. In general, it is observed that the total area conformed by rafts increases with the severity of AD condition (Figure 1). These results can be supported by estimating the number and the size of the low mobility domains following the procedures described in the methodology section. These estimations will be presented below in the prediction section.

### 2.3. Model Validation of Frontal Cortex Lipid Raft Composition

**Figure 2:**
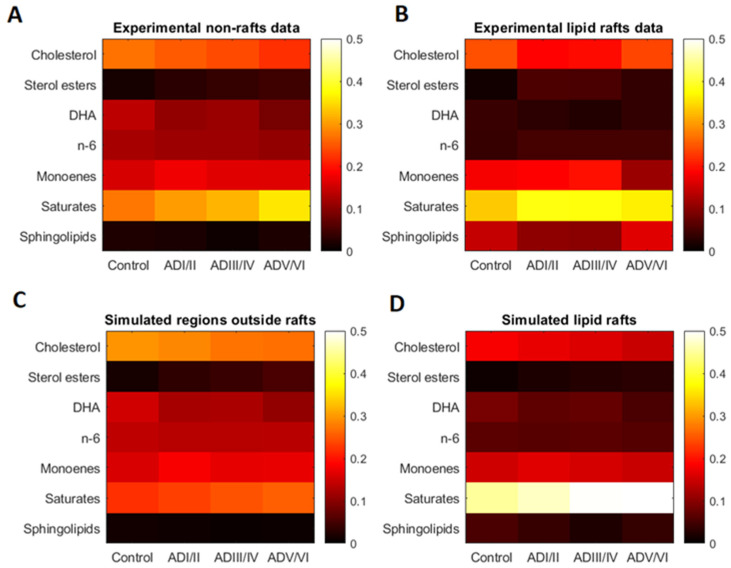
Lipid composition of frontal cortex non-rafts experimental data (**A**), lipid rafts experimental data (**B**), model simulation of regions outside lipid raft (non-rafts) (**D**) and model simulation of lipid raft composition (**C**). Values in color scale indicate proportions to total lipids.

In order to validate the results obtained in the simulation, we next compared the lipid composition of simulated lipid rafts from the model with the composition of frontal cortex lipid rafts summarized in Table 1. Figure 2 presents in panels A and B the actual lipid classes’ composition of cell membranes and lipid rafts from the frontal cortex, respectively. Recall that data in Figure 2A was introduced in the model to perform the simulations to obtain the predicted lipid rafts composition (Figure 2C). As it can be observed, lipid classes were consistently reproduced by the model. Saturated fatty acids and cholesterol simulated proportions in lipid rafts are closer to the experimental measurements of lipid rafts, although the model predicts slightly higher concentrations of saturates in the domains and slightly lower concentrations of cholesterol. The sphingolipids class shows the worst prediction. Sterols and monoenes proportions were not very discriminant for lipid rafts in the experimental data.

**Figure 3:**
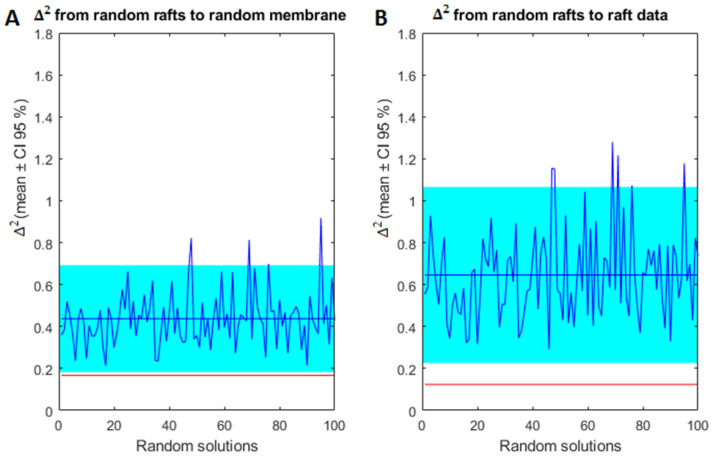
Analysis of results from Figure 2. (**A**) between the random generated cell membrane lipid composition and the corresponding simulated lipid raft composition. (**B**) ∆^2^ between lipid raft compositions presented in panel A and the experimental lipid raft composition (straight blue line represents the mean value and the blue rectangle represents the CI 95%). Red lines indicate the ∆^2^ between the lipid raft composition shown in Figure 2D, and the frontal cortex lipid raft composition (Figure 2B).

Nonetheless, differences between simulated and actual domain composition were detected, which clearly reflect the complexity of membrane lipid organization. Indeed, we did not expect a perfect validation of the model. First, the model is a coarse-grained version of the cell membrane and, more important, the parameters for calibration derived from the familial (genetic) mouse model of AD [12,13], which substantially differs from the sporadic human LOAD [11,14,15]. Another important consideration is that degeneration of nerve cell membranes in human LOAD is not expected to be lineal, but rather is influenced by remodeling mechanisms such as homeoviscous adaptation as the membrane composition changes [11,14,15], which cannot be implemented in our model. Nevertheless, the predictive power of the model may be confirmed by the fact that the validation of the model is significantly different from random results. Thus, results in Figure 3 depict the 95% confidence intervals of quadratic differences (∆^2^) coming from random membrane lipid composition. Specifically, panel A shows the 95% confidence interval of ∆^2^ between random membrane compositions and the corresponding lipid composition of the domain created in each random solution and panel B shows the 95% confidence interval of ∆^2^ between the lipid composition of random simulated rafts and experimental measurements of lipid composition of the domains. The red lines in panels A and B of Figure 3 indicate ∆^2^ for the solution displayed in Figure 2 to compare it with the random solutions. The ∆^2^ between the membrane and the simulated raft using the membrane composition data falls outside the confidence interval in panel A and, also, the difference between the composition of simulated and experimentally measured rafts in the solution of interest is significantly lower than the differences in the random solutions. This observation implies that the validation displayed in Figure 2 is significantly better than a solution obtained introducing a random composition of lipids in the membrane. Based on this validation we used the solutions shown in Figure 2 to predict the number and size of lipid rafts in the different stages of AD.

### 2.4. Prediction of the Number and Size of Lipid Rafts along Stages

**Figure 4:**
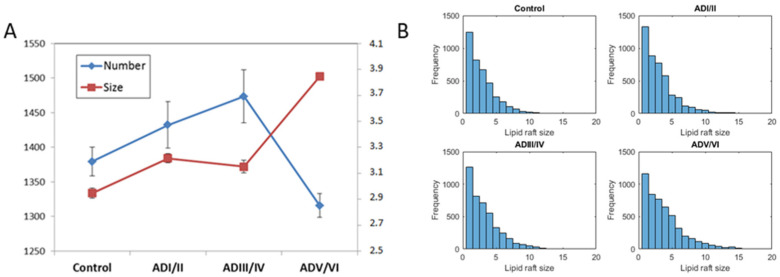
(**A**). Estimation of the number (continuous blue line) and size (dashed red line) of lipid rafts in the four pathological conditions simulated by the model corresponding with the solutions shown Figure 2C. Each point corresponds to the mean ± SD of 10 simulations. (**B**). Frequency histograms of simulated lipid raft sizes.

Differences in raft frequency and size in single solutions after 1000 iterations were suggested in the results from Figure 1. In order to quantify both parameters, we performed quantitative measurement form several simulations of the same solution using the estimators described in the methodology section. The results of these estimators for the number and the size of the simulated lipid rafts in the four neuropathological conditions are shown in Figure 4. It can be observed that the size of raft domains increases in the transition from the control condition to ADI/II, and remain stable up to ADIII/IV stage. Then, raft sizes increase considerably in ADV/VI compared with ADI/II and ADIII/IV. These results are in close agreement with the observations in cortical lipid rafts from transgenic mice [24]. Regarding the number of raft domains, our results predict that the number of rafts increases significantly during earliest stage, reaches a plateau in the intermediate stage, and then decreases dramatically in the latest phase of AD (ADV/VI). These results indicate a reciprocal relationship between raft number and raft size, a finding that may be explained by the existence of a critical point involving domains fusion in advanced AD. This observation is not devoid of pathophysiological consequences as it will be discussed in next section.

### 2.5. Prediction of Membrane Peroxidability during the Evolution of AD

**Figure 5:**
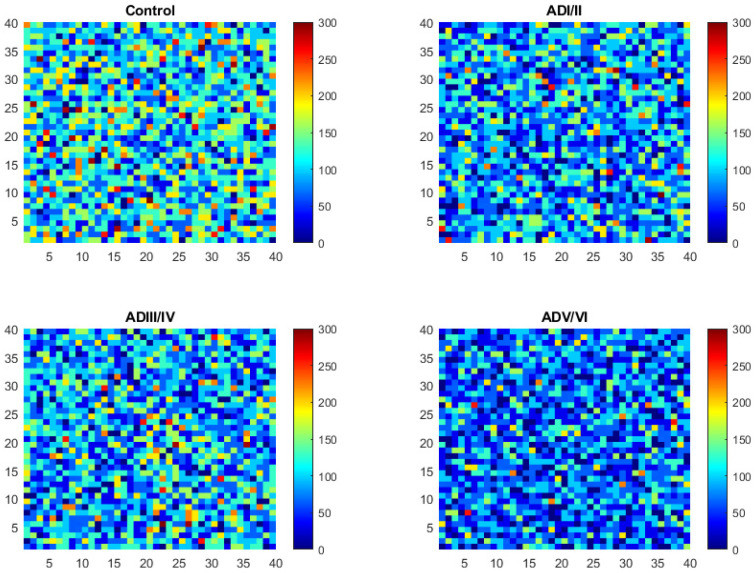
Snapshots of peroxidability index (PI) maps, calculated using formula from [33], in the four neuropathological stages shown in Figure 1. Simulations were obtained after 1000 iterations. Peroxidability index was calculated in 5 × 5 patches from simulations in Figure 1. Color scales represent PI ranges.

Membrane peroxidability is an important factor in the changes of nerve cells during progression of neurodegenerative diseases, including AD. It can be related to the likelihood of nerve cell membranes to respond to endogenous oxidative stress with the generation of reactive lipoperoxides [32]. A peroxidability map calculated from the solution displayed in Figure 1 is shown in Figure 5. These maps were created using Equation (2) using 5 × 5 patches on the original membrane surface. It can be observed that the regions with higher values for PI decrease considerably in AD with respect to control membranes, this pattern is more evident in the last AD phase (ADV/VI).

**Figure 6:**
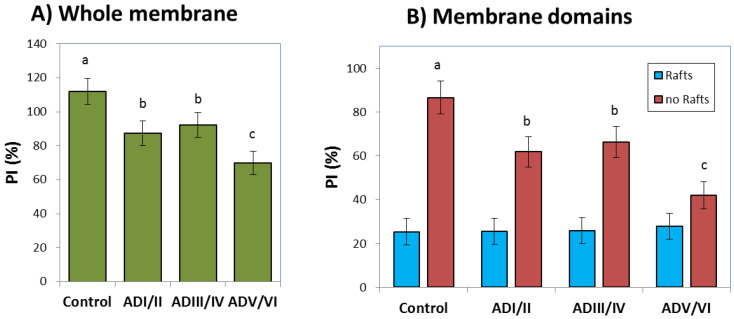
(**A**). Cumulative peroxidability index (PI) in the membrane patches shown in Figure 5. (**B**). Domain peroxidability indexes inside and outside lipid rafts (non-rafts) in the membrane patches shown in Figure 5. Results are expressed as the mean ± SD. Different letters on bars indicate statistically significant differences between groups with *p* < 0.05.

In order to have a deeper insight on the effects of potential changes in AD progression, we have quantified membrane and domain peroxidability indexes (Figure 6). Results from whole membrane patches along the neuropathological sequence (Figure 6A) reveal an early reduction in PI which remains as the disease progresses to the intermediate stage. Furthermore, mean PI calculated in the whole simulated membrane is further decreased in the most advanced stage compared with control and previous stages. This is an important finding, as it suggests that lipid peroxidation might be an early event in the evolution of the disease and that the cellular mechanisms enrolled in antioxidant protection become saturated in the most advanced stage, thereby leading to membrane instability.

Figure 6 also displays PI values calculated inside and outside lipid rafts separately, i.e., lipid rafts and non-raft domains. As expected by the differential composition between lipid rafts and non-rafts under control conditions, PI values in lipid rafts are considerably lower than in non-rafts (Figure 6B). Noticeably, the model predicts that the regions outside lipid rafts are responsible for the significant change in PI observed in whole membranes, as suggested by their identical reduction pattern.

## 3. Discussion

In this work we have used a mathematical model for lipid raft remodeling/formation which has been validated in a transgenic mouse strain of familial AD, to simulate the formation of these microdomains in human brain cortex in different scenarios related with sporadic AD development [24]. The model is a coarse grain version of a molecular dynamic simulation of the cell membrane lipid classes. Lipid classes are approached as cylinders featured by their size and width, to estimate the forces of interaction between the elements of the model by the non-retarded and additive London-Van der Waals forces [34]. Using these approaches and parameters we were able to predict the lipid composition of lipid rafts in the AD mice model with reasonable fidelity [24]. Hence, we found it worthwhile to apply the same mathematical modeling to the human brain cortex. However, a major challenge in this study was that although lipid classes and their proportions were similar between species (mice and human), the pathological causes of the diseases were totally different, i.e., mutated APP/PS1 genes in mice and non-genetic multifaceted in sporadic human AD, both conveyed in the development of amyloid histopathology [35,36,37]. Despite this discrepancy, the prediction made by the model for the lipid composition in microdomains is close to the actual composition, especially for saturated and polyunsaturated fatty acids which are key elements in lipid rafts destabilization during AD [5,38]. Noteworthy, highest similarities with experimental values are observed for most lipid classes during early and intermediate neuropathological stages, a finding that agrees with the notion of homeostatic collapse in overt AD (see below).

As we did not perform any calibration of the parameters, these results can be interpreted as the validation of the mathematical model for the sporadic AD in human cortical membranes. Indeed, the comparison of model results with random parameters shown in Figure 4, revealed significantly better reproduction of actual data than those arising from the modeled lipid composition of microdomains. This checking, on its own, along with the similarity in the lipid profiles between experimental and modeled domains, supports the validity of the simulations performed here on the formation of lipid rafts in human cortex and, more interestingly, reproduce the changes during the neuropathological progression.

Further to simulation of lipid profiles, the current model also allows the estimation of lipid rafts dimensions, namely domain size and number, in the four different scenarios. It is necessary to consider the source of variability in microdomains in the current modeling approach. Regarding domain number, this depends on the initial amount of the small spontaneous aggregations of sphingolipids and cholesterol that act as nucleation points for bigger domains [39]. The formation of the initial spontaneous nucleation points is led by random processes which at the end influences the number of merged domains. On the other side, the average size of the domains is mostly determined by the resulting equilibrium between growth and dissolution of the domains. In turn, the forces between lipid classes affect domain size, depending on the frequency of each lipid class in the membrane.

The prediction that the model makes on the frequency and size of lipid rafts is displayed in Figure 5. Most dramatic changes are the increased proportion of membrane area occupied by lipid rafts domains, and the considerable reduction in its number/frequency, detected in the most advanced stage of AD pathology. It is particularly relevant that the prediction that the increase in lipid raft size observed by the model is mainly attributable to the lipid composition of the cell membrane alone. Another potential factor provoking larger lipid rafts is the coalescence of smaller lipid rafts. This issue is likely answered by the model, as a drastic reduction in raft number occurs simultaneously with the increase in size (Figure 5). Both events have also been predicted for the APP/PS1 model of AD [24], and surely would have enormous consequences for AD proteinopathy. Indeed, this process can lead to increased production of β-amyloid peptides since a significant fraction of APP reside in lipid rafts and their collapse in larger domains will increase APP availability. As γ–secretase protein complex is mostly lipid rafts resident, the higher lipid raft area would provide additional APP for sequential amyloidogenic cleavage, leading to overproduction of amyloid peptide [3,10,40,41]. This observation is in close agreement with our empirical observations in frontal and entorhinal cortex from post-mortem human brains, showing that lipid changes in lipid rafts facilitate APP/BACE1 interactions [5,14,40,42]. Moreover, because β-amyloid can modify the lipid composition of lipid rafts [43,44] and this determines the size of the domains, the increase in the domain size predicted by the mathematical model constitutes a different positive feedback loop by which increased lipid alteration favors additional amyloid generation, extracellular burden and neuronal toxicity, thereby the neuropathology features of AD.

The model also predicts significant changes in the earlier scenarios of ADI/II and ADIII/IV. The prediction indicates an increase in raft number which is not paralleled by changes in size. This interesting observation may be explained by the existence of physiological processes which counteract the physical trend to cause fusion of small lipid rafts, and hence the size-related pathogenicity of amyloid generation. Though there does not exist experimental evidence of such mechanisms in initial stages of AD, it seems that as the disease progresses such homeostatic processes in nerve cells become overloaded and give rise to the progressive increase the number of larger lipid rafts observed in phase V/VI. Additional experimental studies are required to assess the validity of this prediction.

**Figure 7:**
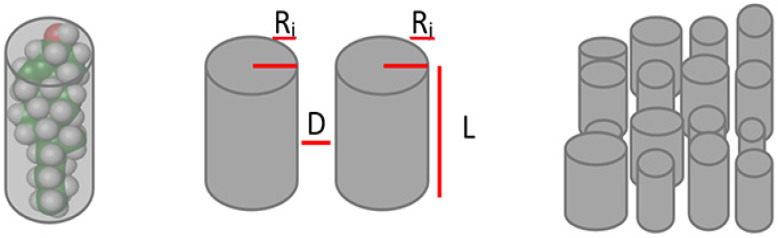
Lipid groups representation. Lipid molecules are simplified as the cylinder that best fits their volume and then are parameterized by their radiuses (Ri, Rj), length (L) and mean distance in between (D). Finally, cylinders are disposed in a 2D matrix at appropriate densities to simulate the cell membrane monolayer [24].

Finally, we have addressed the implications of domain changes in the peroxidability potential. Lipid peroxidation in the brain parenchyma and the local generation of lipid peroxides (LPO) was associated to oxidative stress and cell death in AD [45,46]. In fact, recent studies point to LPO as major inducers of ferroptosis, an iron-dependent mode of apoptosis, which is present in AD-induced cell death [45,47,48]. As shown in Figure 7, our model predicts a reduction in peroxidability in the cell membrane, which is specially marked in the latest stage. When considered separately, non-rafts and lipid rafts undergo stage-dependent reductions of PI, though largest changes occur in non-rafts domains in AD V/VI membranes. As the reduction in PI is concurrent with the depletion in LCPUFA levels (particularly DHA), this involves the concomitant generation of LPO by-products from highly unsaturated fatty acids in non-rafts domains, where LCPUFA were most abundant. Thus, the results from the model suggest a moderate production of reactive lipid peroxides at the expense of PI reduction during initial and intermediate phases. During these stages, deleterious effects of LPO are partially buffered by inducible antioxidant mechanisms, likely driven by oxidation of DHA to hydroxy-2-trans-hexenal (HHE), a reactive aldehyde that signals through Nrf2, as recently demonstrated in nerve cells [16,49,50]. It is in late stages of the disease when metabolic exhaustion [51] impairs adaptive antioxidant mechanisms thus favoring uncontrolled oxidative stress and massive cell death and neurodegeneration.

From one side, this validates the use of the model in humans, and on the other side the validation of the simple model either in the familiar and sporadic AD or between different species; this provides credibility to the lipid raft hypothesis establishing the important role of the lipid composition of the membrane rafts during the evolution of the pathology of AD. Summing up, the mathematical model predicts that lipid rafts of neuronal membranes increase in size due to the change of the lipid composition of cell membranes. This increase in the size of the membrane domains contributes to sustain the evolution of AD and becomes part of a complex positive feedback loop that unbalances the system to a pathological condition.

We are aware of the limitations coming from the coarse-grained nature of our model. However, coarse-grain molecular simulations using agent-based approaches are extremely useful as they allow the simulation of large molecular systems by simplifying the molecular detail of the elements in the model. In our case, we deal with about 40,000 different molecular species distributed within the different lipid classes, with more than 2,000,000 atoms and their corresponding degrees of freedom. Even considering this enormous complexity, the results of the model presented here exhibit a significant degree of reliability as they closely resemble the experimental data previously published in the human frontal cortex [14,15].

## 4. Materials and Methods

### 4.1. Experimental Data

Experimental measurements of lipid composition from frontal cortex cell membrane microdomains (lipid rafts and non-rafts domains) were used to validate the lipid raft model in humans. We collected experimental data from our group in previous publications [11,14,15] and integrated that data together with recent unpublished data from our laboratories including novel lipid classes. The experimental procedures used for the isolation of lipid rafts and non-rafts fractions and to determine their lipid composition is described in [14,15]. Sample sizes for control, AD I/II, AD III/IV, and AD V/VI groups were 19, 12, 15, and 10, respectively.

### 4.2. Mathematical Model

The mathematical model used to simulate membrane dynamics and lipid raft formation is adapted from a previous publication from our group [24]. All the mathematical details of the model are described in this work. Here, we summarize the most important elements of the model. The main framework of the model is a coarse-grain molecular dynamics simulation using an agent-based approach. The model simulates the lipid dynamics in the cell membrane through a piece of monolayer represented by a 200 × 200 matrix. The elements in the matrix are lipid classes aggregated based on structural similarities (Table 2). Each lipid class is represented in the model by a cylinder determined by its width and length (Figure 7). The intermolecular forces between one element and its corresponding four neighbors determine the probability to change its position within the matrix, so that the higher the force between neighbors the lower the probability to switch. At the scale considered in this coarse-grained model, we have neglected the contribution of the water molecules.

Equation (1) is used to estimate the intermolecular forces between the lipid elements of the model based on the parameters from Table 2. This force is estimated through the non-retarded and additive London-Van der Waals (*LVW*) attraction [34,52].

(1) LVWi,j=AL122D32(RiRjRi+Rj)12

Here, *A* is the Hamaker constant, with a value between 10 and 20 and 10 and 19 J (in vacuum). *L* represents the length of each lipid in Å. *D* is the distance between lipids (in Å) whereas *R_i_* and *R_j_* represent the radiuses (Å) of the different lipid classes [52].

Lipid rafts are estimated as regions in the simulated membrane with low mobility, where the mobility the number of changes in position of each element divided by the total number of iterations of the model. Images in Figure 1 represent heatmaps of the mobility of simulated membranes, with the lighter the color, the higher the value of mobility. Darker regions correspond to predicted lipid rafts.

### 4.3. Simulations

The initial condition of the model is created by randomly locating elements from the different lipid classes in the matrix, such that the proportion of elements fits the proportion of lipid classes in frontal cortex membranes experimentally determined in human brains from each neuropathological condition (control, ADI/II, ADIII/IV and ADV/VI) (Figure 2A). Simulations of the model are created by performing interchanges between elements in the matrix based on the calculated probabilities. A single iteration of the model is completed when all the elements of the matrix have had the opportunity to switch once. The model is run for a number of iterations until a steady distribution of low mobility regions is reached. Systematic simulations showed that 1000 iterations were sufficient to reach steady state. After 1000 iterations, the proportion of each lipid class inside the rafts is calculated in the regions with low mobility (black regions in Figure 1). The threshold value for the mobility below which we consider a region in the membrane to be considered a lipid raft was kept from the value fitted with frontal cortex mice data in [24]. In order to deal with stochasticity of the model, the average of 10 simulations is presented as representative of a solution.

As an indirect estimation of simulation noise, we used the root-mean-square deviation (RMSD) of LVW forces that deviates the element positions from their initial location.

Simulations were run in an Intel Core i5-9400F CPU 32 GB RAM using MathWorks Matlab R2020b. As a common rule, the total number of solutions simulated for a single scenario was 10,000.

### 4.4. Analysis of Results

In order to evaluate the significance of the results we used confidence intervals. We first created 100 simulated membranes with random lipid class proportion and predicted from them the proportion of inside rafts. Then, two different measurements were obtained: (i) the quadratic difference (∆^2^) between lipid proportion in the simulated rafts and the corresponding lipid proportion in the simulated membrane and (ii) the quadratic difference between lipid proportion in simulated rafts and the lipid proportion in frontal cortex lipid rafts. From these measurements 95% confidence intervals were estimated. These confidence intervals were then compared with the corresponding measurements i and ii of the simulated lipid rafts obtained using the lipid proportion in frontal cortex membranes. If the simulation using the frontal cortex data falls outside a confidence interval, we will assume there is a significant difference between simulation from random generated membranes and the one obtained from experimental data.

### 4.5. Estimation of Lipid Rafts Number, Lipid Rafts Sizes and Membrane Peroxidability

We estimated the number and size of lipid rafts in a simulated membrane using the same strategy described in [24] from the figures obtained during the simulations. Briefly, the size is estimated by a systematic row-by-row screening of the domains and calculating the average lengths obtained. The number of rafts is estimated from the proportion of membrane occupied by the lipid rafts and the estimation of average lipid size, assuming circular domains.

Membrane peroxidability was assessed through estimation of peroxidability index (*PI*) using Equation (2).

(2) PI=∑% monoenoic·0.025+∑% (n−6 LCPUFA)·5+∑% DHA·6  

*PI* is a linear combination of the mole percentage of unsaturated lipid classes and was adapted from [33] to restrict calculations to the lipid elements in our current model.

## 5. Conclusions

In this work, we have adapted our previous mathematical model for predicting lipid raft formation and evolution from the lipid composition of cell membranes of an AD animal model to sporadic AD in humans. We validate the predictions from the model by comparing the results with the lipid compositions of lipid rafts reported for the human frontal cortex. The model predicts that lipid rafts sizes increase in AD brains and that this increase parallels a reduction in their frequency. Both anomalies correlate with the severity of the disease. We also observed that membrane peroxidability decreases with disease severity, and that most of this reduction occurs in non-raft domains. These predictions might help to better understand the proven links between nerve cell membrane domain destabilization and the early events leading to AD.

## Figures and Tables

**Table 1:** Lipid class composition of frontal cortex cell membrane and lipid rafts from experimental measurements. Values are expressed as % of total lipid class.

	(A) Frontal Cortex Lipid Rafts				(B) Frontal Cortex Cell Membrane			
	Control	ADI/II	ADIII/IV	ADV/VI	Control	ADI/II	ADIII/IV	ADV/VI
Cholesterol	24.57	18.87	19.40	23.78	27.06	25.24	24.05	22.20
Sterol esters	1.20	5.57	5.33	3.69	1.58	2.96	3.81	4.51
DHA	4.28	3.16	2.42	3.66	13.73	10.66	11.16	8.68
n-6 LCPUFA	3.95	4.95	4.90	4.89	12.19	11.41	11.49	10.69
Monoenes	18.01	18.71	19.81	11.31	15.71	17.72	16.53	16.29
Saturates	33.54	38.26	38.03	36.18	27.47	30.10	31.90	35.67
Sphingolipids	14.45	10.49	10.10	16.49	2.26	1.92	1.06	1.97

**Table 2:** Model parameters estimated from mice frontal cortex membranes [24] to represent lipid classes as cylinders in the agent-based model.

	Width (Radius Å)	Length (Å)
Cholesterol	5.94	19.99
Sterol esters	5.94	19.99
DHA	6.74	19.01
n-6 LCPUFA	5.84	19.70
Monoenoic fatty acids	5.30	22.59
Saturated fatty acids	6.14	22.44
Sphingolipids	6.18	22.44
Intermolecular distance (Å)	1.50	
